# Effect of tolvaptan on renal involvement in patients with autosomal dominant polycystic kidney disease according to different gene mutations

**DOI:** 10.1007/s10157-020-01988-4

**Published:** 2020-11-03

**Authors:** Tomofumi Moriyama, Yosuke Nakayama, Mikiko Soejima, Yunosuke Yokota, Kanji Ota, Sakuya Ito, Goh Kodama, Nao Nakamura, Yuka Kurokawa, Junko Yano, Utako Ueda, Yoshimi Takamiya, Yusuke Kaida, Takuma Hazama, Ryo Shibata, Yoshiro Koda, Kei Fukami

**Affiliations:** 1 Division of Nephrology, Department of Medicine, Kurume University School of Medicine, 67 Asahi-machi, Kurume city, Fukuoka Japan; 2 Department of Forensic Medicine, Kurume University School of Medicine, Kurume, Japan

**Keywords:** Autosomal dominant polycystic kidney disease, Tolvaptan, PKD1, PKD2, Renal function, Total kidney volume

## Abstract

**Background:**

Autosomal dominant polycystic kidney disease (ADPKD) is an inherited disorder caused by mutations in the polycystic kidney disease (*PKD*) gene. Although tolvaptan has benefits for renal involvement, the different effects depending on the gene mutation type are unknown. Thus, we explore the different effects of tolvaptan on the annual changes in total kidney volume (%TKV) and estimated glomerular filtration rate (eGFR) according to the gene mutation type in ADPKD patients.

**Methods:**

In total, 135 ADPKD patients were screened, and 22 patients taking tolvaptan for at least a year were retrospectively studied at the Kurume University Hospital. We examined the decline in renal function and %TKV by computed tomography and analyzed the gene mutation. Patients were classified into the following four groups according to gene mutation type: *PKD1*-truncated, *PKD1-*non-truncated, *PKD2*, and mutation not found. Patients were treated with tolvaptan, and the effects of tolvaptan were analyzed according to the gene mutation type.

**Results:**

Patients (age: 52.3 ± 11.2 years) were administered tolvaptan at a dose of 45 or 60 mg. No variation was observed in the annual changes in eGFR (%eGFR) (before: − 10.5% ± 13.9%, after: − 14.4% ± 8.1%, *P* = 0.139), whereas %TKV was significantly improved after the tolvaptan treatment (before: 14.9% ± 8.0%, after: − 5.4% ± 7.6%, *P* < 0.001). Unlike %eGFR, tolvaptan treatment significantly improved %TKV, regardless of the type of gene mutation.

**Conclusions:**

A year treatment with tolvaptan significantly improved %TKV in patients with ADPKD, regardless of the gene mutation type.

**Electronic supplementary material:**

The online version of this article (10.1007/s10157-020-01988-4) contains supplementary material, which is available to authorized users.

## Introduction

Autosomal dominant polycystic kidney disease (ADPKD) is the most commonly inherited kidney disorder, and almost 31,000 patients are diagnosed with ADPKD in Japan [1]. Approximately 85% of the patients have polycystic kidney disease (*PKD*)1 (16q13.3) gene mutation, and the remaining patients have *PKD2* (4q21) gene mutation [2]. ADPKD is caused by the dysregulation of protein polycystin 1 (PC1) or protein polycystin 2 (PC2) coded by the *PKD* gene on tubular epithelial cells, which could decrease the Ca^2+^ inflow into the cells and increase cAMP production, leading to renal cyst growths [3]. Recently, the use of tolvaptan, a vasopressin type 2 receptor antagonist, has spread worldwide as a treatment for ADPKD, and recent large clinical studies clearly demonstrated the effects of tolvaptan in alleviating renal cyst growths and the estimated glomerular filtration rate (eGFR) decrease in these patients [4, 5].

The difference in the genetic background has been reported to affect the renal prognosis in ADPKD patients. Indeed, the patients with *PKD1* gene mutation whose condition progressed to end-stage renal failure were younger than those with *PKD2* gene mutation [6], and the renal prognosis and survival rate of patients with *PKD1*-truncating gene mutation (*PKD1-T*) were worse than those of patients with other gene mutations [7]. This suggests that the gene analysis for ADPKD is useful for predicting the prognosis. However, variations in the effects of tolvaptan on renal cyst growth and renal function in patients with ADPKD based on the gene mutations remain obscure. This study aimed to explore the different effects of tolvaptan on the annual changes in total kidney volume (%TKV) and eGFR according to the type of gene mutation in patients diagnosed with ADPKD.

## Materials and methods

### Patients and study design

**Fig. 1:**
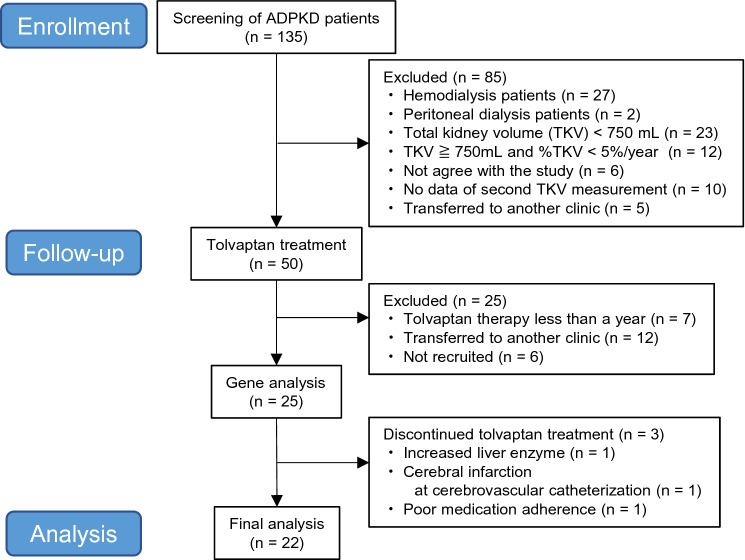
Flowchart of the study. *ADPKD* autosomal dominant polycystic kidney disease, *TKV* total kidney volume

From January 1998 to May 2019, a total of 135 ADPKD patients were screened, and 50 patients received tolvaptan treatment at Kurume University Hospital; of these, 22 patients who were taking tolvaptan for at least a year were analyzed in this study (Fig. 1). Sixteen patients could be followed up for 2 years. Almost all patients were diagnosed with ADPKD and met the indication criteria for tolvaptan treatment according to the insurance adaptation in Japan (TKV ≥ 750 mL, %TKV/year ≥ 5%, and eGFR ≥ 15 mL/min/1.73 m^2^). The medical history was obtained with a questionnaire. After fasting, blood samples were drawn from the antecubital vein to determine the patients’ laboratory data, including serum creatinine (Cr) and uric acid, which were measured at a commercially available laboratory (Daiichi Pure Chemicals Co., Ltd, Tokyo, Japan). The other laboratory data were measured at a different commercially available laboratory (Wako Pure Chemical Industries, Ltd, Osaka, Japan). Spot urine was collected for the determination of proteinuria (g/gCr). eGFR was calculated using the following formula: eGFR = 0.741 × 175 × serum Cr − 1.154 × age − 0.203 × (0.742 if female) [8, 9]. %TKV and eGFR before and after treatment were calculated using the following formula: (post-data − pre-data)/pre-data × 100 (%). Therefore, the initial drop in eGFR was included in the %eGFR. Patients were retrospectively examined, and 16 patients could be followed up for 2 years. Renal function and TKV determined by computed tomography (CT) were evaluated yearly. Furthermore, a gene analysis was performed in 22 ADPKD patients. Informed consent was obtained from all patients, and the study protocol was approved by the Ethical Committee of Kurume University (Ethics No. 304). This work was conducted in accordance with the Declaration of Helsinki and was registered in the University Hospital Medical Information Network clinical trials database (UMIN: 000037987).

### Measurement of the total kidney volume

TKV was measured by CT using the volumetric methods. Briefly, a radiologist was blinded to the measurements collected by the nephrologist. Axial CT images acquired at a 5-mm slice thickness were electronically transferred to the imaging workstation. The renal cortex was traced on each image showing renal parenchyma, and the renal pelvis was excluded from the volume measurement. Following complete tracing of each kidney, TKV was automatically calculated using the volume measurement software available on Ziostation 2 (Ziosoft, Inc., Tokyo, Japan), SYNAPSE VINCENT (FUJIFILM, Inc., Tokyo, Japan), or AZE Virtual Place (Canon, Inc., Tokyo, Japan).

### Genetic analysis

Genomic DNA from the peripheral blood was prepared using the Gentra Puregene Cell Kit (QIAGEN, Venlo, The Netherlands). Sequencing analysis was performed by either next-generation sequencing (NGS) or Sanger sequencing. NGS of *PKD1* (*PKD1* truncating mutation: *PKD1-T*, *PKD1* non-truncating mutation: *PKD1-NT*) and *PKD2* was performed by the Kazusa DNA Research Institute (NextSeq500, Illumina), and variants with a frequency of < 0.1% or variants already reported to be disease-associated mutations were reported. To perform the Sanger sequencing, LR-PCR primers were used to amplify 18 long DNA fragments, including the exonic regions of *PKD1* and *PKD2* [10]. The amplified fragments were directly sequenced using PCR primers, and the sequence primers were synthesized [11, 12]. The SeqScape Software 3 v3.0 (Thermo Fisher Scientific Inc.) was used to detect mutations based on comparisons with reference sequences. We could not identify any mutation in both *PKD1* and *PKD2* in 4 patients and, therefore, described their mutation type as “mutation not found” in this study. The identified mutations were checked for their registration on Autosomal Dominant Polycystic Kidney Disease: Mutation Database (https://pkdb.mayo.edu/cgi-bin/v2_display_mutations.cgi?apkd_mode=PROD). The pathogenicity of a missense mutation, 4550A > C identified in Case 11 that has not been previously identified, was predicted using Mutation Taster (https://www.mutationtaster.org/) to be “disease-causing”. In addition, this mutation might be rare because it is not found in public databases, such as 1000 genome data (https://www.internationalgenome.org/home); therefore, it was considered a “*PKD1-NT*” mutation in this study. The other novel mutations, such as frameshift or nonsense mutations, were regarded as pathogenic.

### Statistical analysis

A paired *t*-test was performed to compare %eGFR and %TKV before and after the treatment. All statistical analyses were performed using JMP Pro ver. 14 Software (SAS Institute Inc.). Data are shown as mean ± standard deviation, and a *p* value of < 0.05 was considered statistically significant.

## Results

### Clinical characteristics and gene analysis of the patients

**Fig. 2:**
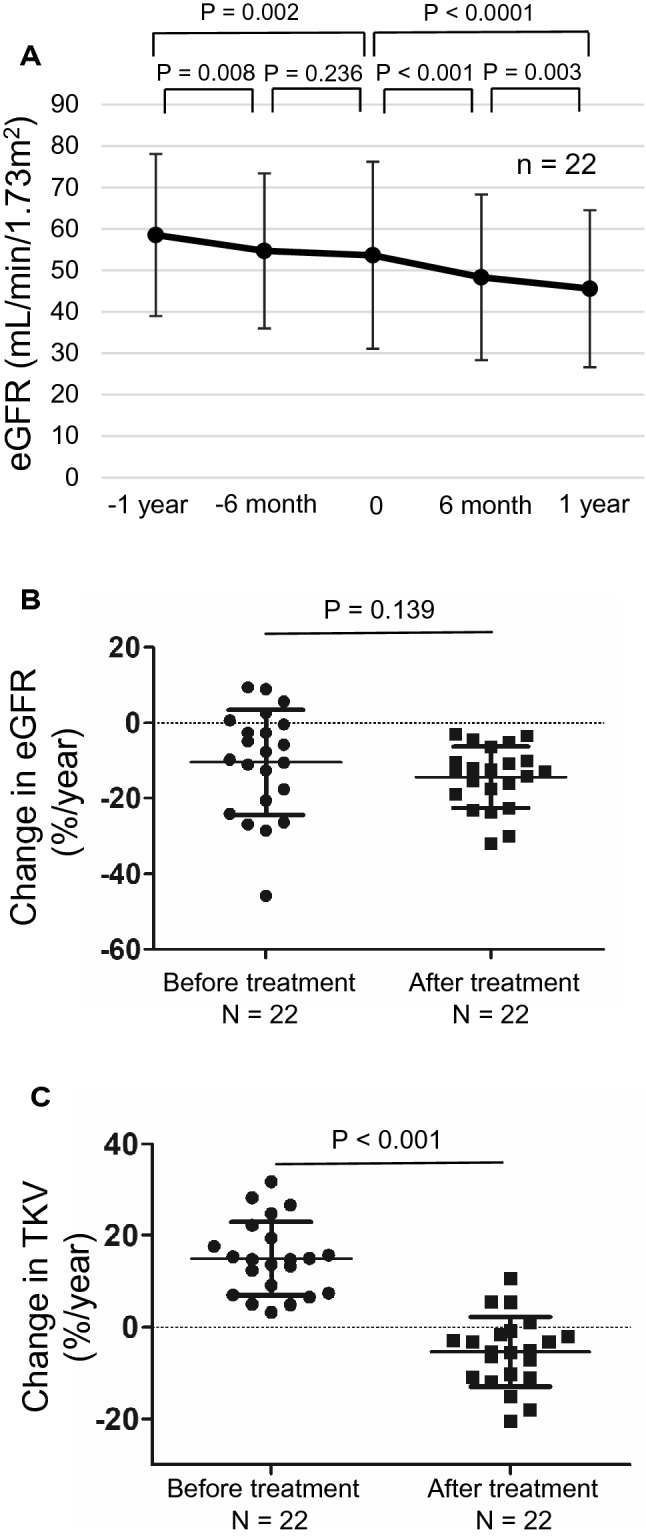
Effects of tolvaptan on eGFR and change in eGFR and TKV. **a** eGFR levels at 1 year before and at 1 year after the tolvaptan treatment (*n* = 22). **b** Change in eGFR at 1 year before to at 1 year after the tolvaptan treatment (*n* = 22). **c** Change in TKV at 1 year before to at 1 year after the tolvaptan treatment (*n* = 22). *eGFR* estimated glomerular filtration rate, *TKV* total kidney volume

The clinical characteristics of the patients just before the tolvaptan treatment is shown in Table 1. Patients’ mean age and eGFR were 52.3 ± 11.2 years and 53.6 ± 22.6 ml/min/1.73 m^2^, respectively. The total mean TKV was 1976 ± 1142 mL, and the mean proteinuria level was 0.27 ± 0.44 g/gCr. A family history of ADPKD was observed in 13 patients (59%) (Table 1). Gene analysis demonstrated *PKD1-T* (*n* = 10), *PKD1-NT* (*n* = 3), *PKD2* mutation (*n* = 5), and mutation not found (*n* = 4) in 22 ADPKD patients (Table 2). Almost all patients, except for two patients, had liver cysts (91%). The prevalence of unruptured cerebral aneurysm (UCA) and subarachnoid hemorrhage (SAH) was 32% (seven in 22 patients). The prevalence of UCA and SAH in patients with *PKD1* gene mutation was 54% (seven in 13 patients), whereas none of the patients with *PKD2* and mutations not found had UCA and/or SAH (Table 2). %TKV before treatment was not affected by the type of gene mutation (data not shown). In all patients, %eGFR significantly decreased, regardless of tolvaptan treatment (a year before treatment: 58.5 ± 19.5 mL/min/1.73 m^2^, just before treatment: 53.6 ± 22.6 mL/min/1.73 m^2^, a year after treatment: 45.6 ± 18.9 mL/min/1.73 m^2^, a year before treatment vs. just before treatment: *P* = 0.002, just before treatment vs. a year after treatment: *P* < 0.0001) (Fig. 2a). No difference in the decline in %eGFR was observed before and after the treatment (before: − 10.5% ± 13.9%, after: − 14.4% ± 8.1%, *P* = 0.139) (Fig. 2b), whereas annual %TKV was significantly reduced by the tolvaptan treatment (before: 14.9% ± 8.0%, after: − 5.4% ± 7.6%, *P* < 0.001) (Fig. 2c).

**Table 1:** Clinical characteristics of the patients just before the tolvaptan treatment

No. of patients	22
Age (years)	52.3 ± 11.2
No. of male patients (%)	11 (50)
SBP (mmHg)	134 ± 14
DBP (mmHg)	84 ± 12
Hemoglobin (g/dL)	13.2 ± 1.8
Hematocrit (%)	38.8 ± 4.3
Total protein (g/dL)	6.95 ± 0.42
Serum albumin (g/dL)	4.12 ± 0.36
AST (IU/L)	21.2 ± 8.3
ALT (IU/L)	19.5 ± 13.1
γ GTP (IU/L)	46.5 ± 45.3
BUN (mg/dL)	20.4 ± 6.6
Serum Cr (mg/dL)	1.15 ± 0.44
eGFR (mL/min/1.73 m^2^)	53.6 ± 22.6
Proteinuria (g/gCr)	0.27 ± 0.44
Hematuria (%)	5 (23)
Uric acid (mg/dL)	6.1 ± 1.4
TKV (mL)	1976 ± 1142
%TKV (%/year)	14.9 ± 8.0
RAS-i use (%)	16 (73)
Family history ( +) (%)	13 (59)

Values are shown as mean ± standard deviation

*No.* number, *SBP* systolic blood pressure, *DBP* diastolic blood pressure, *AST* aspartate aminotransaminase, *ALT* alanine aminotransaminase, *γ GTP* γ glutamyl transpeptidase, *BUN* blood urea nitrogen, *Cr* creatinine, *eGFR* estimated glomerular filtration rate, *TKV* total kidney volume, *RAS-i* renin angiotensin system inhibitor

**Table 2:** Gene mutations and characteristics of the patients

Case	Age	Sex	Gene mutation	Exon	DNA change	Protein change	eGFR (mL/min/1.73 m^2^)	TKV (mL)	ADPKD family history	Liver cyst	UCA or SAH
1	39	M	PKD1 T	IVS43	12,004-1G > A	A4002fs	52.2	2045	+	+	−
2	45	F	PKD1 T	15	4625dupT	T1543fs	71	916	+	+	+
3	62	M	PKD1 T	15	5379delC	V1794fs	46.1	2403	+	+	−
4	64	F	PKD1 T	46	12684_12685insG	N4229fs	39.2	1431	−	+	+
5	52	F	PKD1 T	4	482_500del19-fs-*	L161fs122X	38.8	844	−	+	+
6	45	F	PKD1 T	IVS45	12,445-3delCA	E4149fs	25	2385	+	+	+
7	40	M	PKD1 T	15	4447C > T	Q1483X	80.4	1256	−	−	+
8	57	M	PKD1 T	40	11343C > A-*	Y3781X	46	2628	+	+	+
9	68	F	PKD1 T	15	6379_6380delTC	S2127fs	20.5	4064	−	+	+
10	43	M	PKD1 T	15	4551C > A	Y1517X	34.4	3767	−	+	−
11	70	F	PKD1 NT	15	4550A > C*	Y1517S	33.2	1296	+	+	−
12	39	F	PKD1 NT	15	6397_6399delTTC	F2133del	31.5	3000	+	+	−
13	30	M	PKD1 NT	40	11340_11345delTTACGA	Y3781_D3782del2	82	1400	+	+	−
14	55	F	PKD2	1	316delG fs-*	E106fs10X	55.7	1754	+	+	−
15	55	F	PKD2	6	1525_1529del5 ins15-fs-*	C509fs20X	87.3	1026	+	+	−
16	66	M	PKD2	4	1081C > T	R361X	49.6	1510	+	+	−
17	43	M	PKD2	4	958C > T	R320X	76.8	1084	−	+	−
18	63	M	PKD2	4	1081C > T	R361X	45.5	5130	+	+	−
19	58	M	Not found				40.9	901	−	−	−
20	50	F	Not found				55.8	1126	+	+	−
21	44	F	Not found				110	1565	−	+	−
22	62	M	Not found				58.1	1931	−	+	−

*No.* number, *eGFR* estimated glomerular filtration rate, *TKV* total kidney volume, *ADPKD* autosomal dominant polycystic kidney disease, *UCA* unruptured cerebral aneurysm, *SAH* subarachnoid hemorrhage, *PKD1-T* polycystic kidney disease1 truncating mutation, *PKD1-NT* polycystic kidney disease1 non-truncating mutation, *PKD2* polycystic kidney disease2

*Novel gene mutation

### Effect of tolvaptan on %eGFR and %TKV according to the PKD gene mutation

**Fig. 3:**
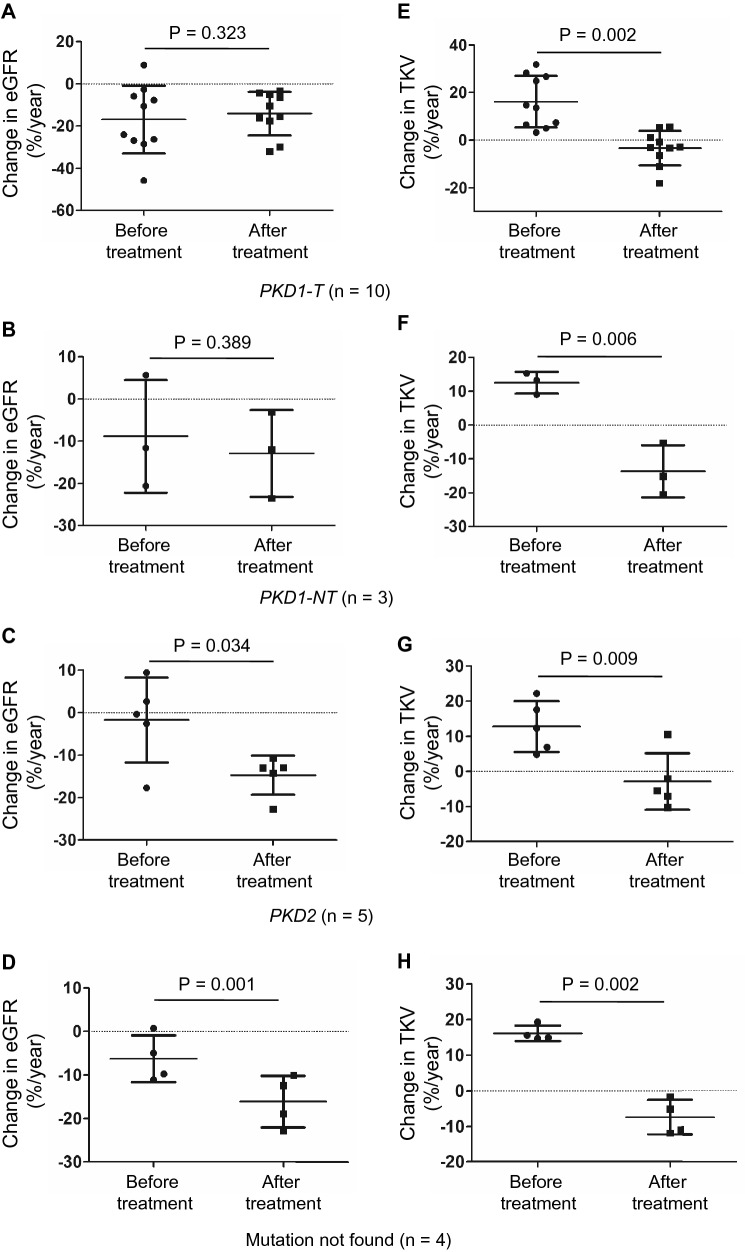
Effects of tolvaptan on changes in eGFR and TKV according to the type of gene mutation. Change in eGFR from at 1 year before to at 1 year after the tolvaptan treatment in patients with *PKD1-T* (**a**), *PKD1-NT* (**b**), *PKD2* (**c**), or mutation not found. Change in TKV from at 1 year before to at 1 year after the tolvaptan treatment in patients with *PKD1-T* (**e**), *PKD1-NT* (**f**), *PKD2* (**g**), or mutation not found. *eGFR* estimated glomerular filtration rate, *TKV* total kidney volume, *PKD1-T* polycystic kidney disease1-truncating gene mutation, *PKD-NT* polycystic kidney disease-non-truncating gene mutation

%eGFR did not change before and after the treatment in patients with *PKD1*-*T* and *PKD1*-*NT* gene mutations (before: –16.9% ± 16.1%, after: –14.1% ± 10.3%, P = 0.323, before: − 8.8% ± 13.4%, after: − 12.9% ± 10.3%, *P* = 0.389, respectively) (Figs. 3a, b). However, in patients with *PKD2* and mutations not found, %eGFR showed a significant decrease (before: − 1.7% ± 10.0%, after: − 14.7% ± 4.6%, *P* = 0.034; before: − 6.3% ± 5.4%, after: − 16.1% ± 5.9%, P = 0.001, respectively) (Figs. 3c, d). On the contrary, %TKV was significantly ameliorated by the tolvaptan treatment regardless of the type of gene mutation (before: 16.2% ± 10.8%, after: − 3.3% ± 7.2%, *P* = 0.002; before: 12.5% ± 3.2%, after: − 13.6% ± 7.7%, *P* = 0.006; before: 12.8% ± 7.2%, after: − 2.8% ± 8.0%, *P* = 0.009; before: 16.2% ± 2.2%, after: − 7.4% ± 4.9%, *P* = 0.002, respectively) (Fig. 3e–h). Annual %eGFR was significantly improved in patients who underwent 2-year treatment compared with those who received 1-year treatment. (Online Resource 1A). The decrease in annual %TKV by 1-year tolvaptan treatment returned to baseline in patients with 2-year treatment; however, it was still significant compared with %TKV at baseline independent of tolvaptan dose (Online Resource 1B and C). Both annual %eGFR and %TKV returned to baseline levels; however, the benefits of 2-year tolvaptan treatment on annual %TKV were sustained in 16 patients, regardless of the gene mutation type (Online Resource 2A-H).

### Dose of tolvaptan and adverse events

**Fig. 4:**
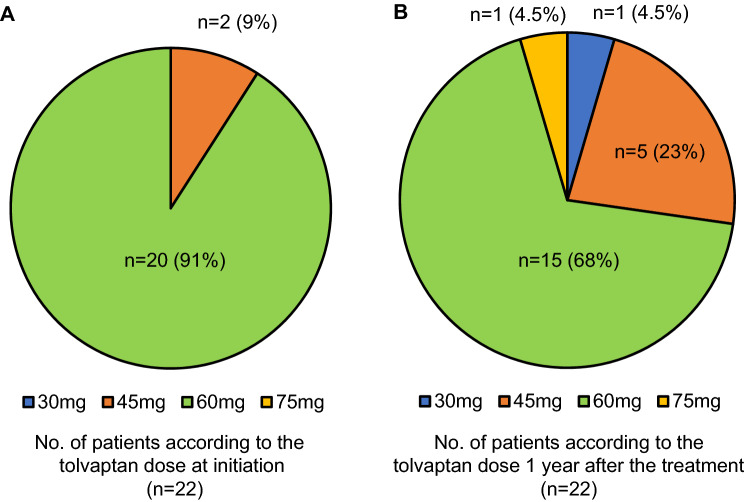
Dose of tolvaptan at initiation and at 1 year after the tolvaptan treatment

In total, 20 patients (91%) were administered tolvaptan at a dose of 60 mg, according to the manufacturer’s instruction (Otsuka Pharmaceutical Co., Ltd.). Tolvaptan at a dose of 45 mg was initiated in the remaining patients (*n* = 2) (9%) because of polyuria (Fig. 4a). The mean dose of tolvaptan did not change at the initiation of and at 1 year after the treatment (58.6 ± 4.4, 55.9 ± 9.5 mg/day, *P* = 0.081). The tolvaptan dose was increased to 75 mg in one patient, whereas, in four patients, the dose was reduced (18%) (Fig. 4b) because of increased liver enzyme levels (*n* = 1), insufficient water intake (*n* = 1), polyuria (*n* = 1), and hypernatremia (*n* = 1). The mean dose of tolvaptan after the 2-year treatment was 63.3 ± 21.7 mg/day (*n* = 16).

## Discussion

In this study, we demonstrated that the 1-year treatment with tolvaptan significantly reduced annual %TKV in Japanese ADPKD patients, regardless of the type of gene mutation. This positive effect was sustained for 2 years without a maximal dose of tolvaptan treatment.

Torres et al. examined the effects of tolvaptan on ADPKD-induced renal involvement in their TEMPO 4:4 study and demonstrated that the treatment with tolvaptan significantly improved the annual %TKV in patients with both *PKD1-T* and *PKD1-NT/PKD2* gene mutations compared with the placebo-treated group [13]. In this study, the 1-year treatment with tolvaptan significantly decelerated the annual TKV growth not only in patients with *PKD1-T, PKD1-NT*, and *PKD2* gene mutations, but also in those with mutation not found. This suggests that tolvaptan might exert an inhibitory effect on cyst growth in patients with mutation not found. Recently, in addition to *PKD1* and *PKD2*, *GANAB* (11q12.3) and *DNAJB11* gene mutations have been reported to play a pathogenic role in ADPKD development [14, 15], and these mutations could downregulate PC1 expression in tubular cells. These findings suggest that our mutation not found patients might have these gene mutations, leading to cyst growth and renal dysfunction. Furthermore, in the TEMPO 4:4 study, Japanese patients were excluded from the study, and the Asian patients only accounted for 0.6% of all enrolled patients [13]. Therefore, the present study is the first report showing that tolvaptan has protective effects on TKV growth in Japanese ADPKD patients, regardless of the type of gene mutation.

%eGFR at the end of the first year of treatment was decreased in patients with *PKD2* gene mutation and mutation not found. In contrast, it was unchanged in patients with *PKD1-T* and *PKD1-NT* gene mutations in this study. The effect of tolvaptan on renal function is reportedly dependent on the baseline renal function [16]. Tolvaptan significantly delayed the progression of renal function in Chronic Kidney Disease (CKD) stage 2 and CKD stage 3 patients, unlike in CKD stage 1 patients. In our patients with *PKD2* gene mutation and mutation not found, the baseline eGFR was higher, and the annual %eGFR decline at baseline seemed slower than those with other gene mutations (Table 3). This may explain why our patients with *PKD2* gene mutation and mutation not found showed more decline in eGFR with the tolvaptan treatment, although the %eGFR in these patients normalized after 2 years of tolvaptan treatment. However, because of the small sample size, this seems still inconclusive yet.

**Table 3:** Clinical characteristics of the patients according to the PKD gene mutation at tolvaptan introduction

	PKD1-T	PKD1-NT	PKD2	Mutation not found
No. of patients	10	3	5	4
Age (years)	51.5 ± 10.6	46.3 ± 21.0	56.4 ± 8.9	53.5 ± 8.1
Sex (male)	5 (50)	1 (33)	3 (60)	2 (50)
SBP (mmHg)	133 ± 12	137 ± 21	129 ± 19	139 ± 12
DBP (mmHg)	79 ± 9	101 ± 11	83 ± 16	88 ± 5
Hemoglobin (g/dL)	12.8 ± 2.2	13.5 ± 1.7	13.9 ± 1.6	12.9 ± 0.8
Hematocrit (%)	37.2 ± 5.0	40.4 ± 4.4	41.1 ± 3.3	38.4 ± 2.6
Total protein (g/dL)	6.98 ± 0.41	7.02 ± 0.40	6.96 ± 0.61	6.82 ± 0.32
Serum albumin (g/dL)	4.09 ± 0.39	4.18 ± 0.35	4.17 ± 0.49	4.11 ± 0.22
AST (IU/L)	18.1 ± 4.4	17.7 ± 2.1	22.8 ± 5.6	29.5 ± 15.5
ALT (IU/L)	14.4 ± 4.3	16.7 ± 11.7	22.8 ± 11.3	30.0 ± 24.9
γ GTP (IU/L)	44.1 ± 43.8	23.0 ± 11.1	46.0 ± 34.1	70.5 ± 75.3
BUN (mg/dL)	23.6 ± 7.0	20.9 ± 2.9	15.8 ± 4.5	17.6 ± 7.1
Serum Cr (mg/dL)	1.32 ± 0.42	1.23 ± 0.32	0.94 ± 0.29	0.95 ± 0.40
eGFR (mL/min/1.73m^2^)	45.4 ± 18.8	48.9 ± 28.7	63.0 ± 18.2	66.2 ± 30.2
Proteinuria (g/gCr)	0.27 ± 0.37	0.13 ± 0.06	0.18 ± 0.31	0.47 ± 0.85
Hematuria (%)	1 (10)	1 (33)	1 (20)	2 (50)
Uric acid (mg/dL)	6.5 ± 1.4	5.8 ± 1.2	6.0 ± 1.5	5.2 ± 1.4
TKV (mL)	2174 ± 1114	1899 ± 955	2101 ± 1720	1381 ± 459
%TKV (%/year)	16.2 ± 10.8	12.5 ± 3.2	12.8 ± 7.2	16.2 ± 2.2
RAS-i use (%)	8 (80)	1 (33)	5 (100)	2 (50)
Family history (%)	5 (50)	3 (100)	4 (80)	1 (25)

Values are shown as mean ± standard deviation or range

*PKD* polycystic kidney disease, *PKD1-T* polycystic kidney disease1-truncated gene mutation, *PKD-NT* polycystic kidney disease1-non-truncated gene mutation, *No.* number, SBP systolic blood pressure, *DBP* diastolic blood pressure, *AST* aspartate aminotransaminase, *ALT* alanine aminotransaminase, *γ GTP* γ glutamyl transpeptidase, *BUN* blood urea nitrogen, *Cr* creatinine, *eGFR* estimated glomerular filtration rate, *TKV* total kidney volume, *RAS-i* renin-angiotensin system inhibitor

Interestingly, the prevalence of UCA and SAH was higher in our patients than in the other previously reported cohort [17]. Furthermore, UCA and SAH occurred in patients with *PKD1-T* and *PKD1-NT* gene mutations, whereas no history of UCA and SAH was found in patients with *PKD2* and mutation not found. To date, only a few studies have shown the relationship between *PKD* gene mutations and the prevalence of brain aneurysms. Rossetti et al. reported the risk of a brain aneurysm in patients with *PKD1* and *PKD2* gene phenotypes in ADPKD patients [18]. The difference in the prevalence of UCA and SAH, according to the different types of gene mutations, in this study might be due to the different races and backgrounds of the enrolled patients. Thus, further research is warranted to clarify this issue.

In large clinical studies, oral tolvaptan was administered in daily split-dose regimens of 45/15, 60/30, or 90/30 mg, and approximately half of the patients were prescribed a dose of 120 mg; their data showed that the effect of tolvaptan on renal involvement was dose-dependent [4, 5]. However, in our study, regardless of whether the dose of tolvaptan was ≤ 60 mg, annual %TKV was significantly reduced. In the TEMPO3:4 study, the patients’ mean height and body weight were 1.75 ± 0.10 m and 80.5 ± 17.7 kg, respectively, whereas these were much lower (1.65 ± 0.08 m and 66.2 ± 11.2 kg, respectively) in our patients. Therefore, tolvaptan might be effective if administered at a low dose, as previously reported, and could be adjusted according to the physique of ADPKD patients.

The most concerning side effect of tolvaptan is the increase in the liver enzyme. In a study that examined its drug safety in Japanese ADPKD patients, patients with increased liver enzyme levels after tolvaptan treatment could be re-administered tolvaptan after treatment suspension; however, the tolvaptan dose has to be reduced briefly [19, 20]. In this study, two of 50 patients treated with tolvaptan had increased liver enzyme levels, which was similar to a previous report [16]. One patient showed a slight increase in the liver enzyme level; thus, we reduced the dose of tolvaptan from 60 to 45 mg, and the patients’ liver enzyme levels normalized after changing the dosage. This patient could continue with the tolvaptan treatment. The liver enzyme level in the other patient was three times higher; thus, we discontinued the treatment. The prevalence of an increase in liver enzyme was 4% in this study, which was similar to the previous report, though study duration was different [16].

There are several limitations in this study. First, the small sample size was insufficient for analysis and rendered it difficult to draw definite conclusions from this study. Second, we could not detect *PKD* gene mutations, including large insertion or deletion, in patients with mutation not found. Third, the study period is short. Finally, the tolvaptan dose in this study was quite low and, thus, might not be adequate to confirm the benefits of tolvaptan. Therefore, further large and longitudinal clinical studies with a precise gene analysis and high administered tolvaptan dose are warranted to verify the efficacy of tolvaptan on renal involvement in ADPKD patients according to the different types of gene mutations.

## Conclusion

In this study, tolvaptan treatment for a year significantly improved annual %TKV in patients with ADPKD, regardless of the type of gene mutation.

## Electronic supplementary material

Below is the link to the electronic supplementary material.

Supplementary file1 (PDF 207 kb)
