# Supplementary material for: Effect of tolvaptan on renal involvement in patients with autosomal dominant polycystic kidney disease according to different gene mutations
Source: Clin Exp Nephrol. 2020 Nov 3;25(3):251–60. doi: 10.1007/s10157-020-01988-4 (PMC7925507; doi:10.1007/s10157-020-01988-4)
Supplement: Supplementary file 1 — Supplementary file1 (PDF 207 kb) [file 10157_2020_1988_MOESM1_ESM.pdf]

# Supplemental Figures

Title: Effect of tolvaptan on renal involvement in patients with autosomal dominant polycystic kidney disease according to the different gene mutation

Tomofumi Moriyama[1], Yosuke Nakayama[1], Mikiko Soejima[2], Yunosuke Yokota[1], Kanji Ota[1], Sakuya Ito[1], Go Kodama[1], Nao Nakamura[1], Yuka Kurokawa[1], Junko Yano[1], Utako Ueda[1], Yoshimi Takamiya[1], Yusuke Kaida[1], Takuma Hazama[1], Ryo Shibata[1], Yoshiro Koda[2], Kei Fukami[1]

[1]Division of Nephrology, Department of Medicine, Kurume University School of Medicine, Kurume, Japan

[2]Department of Forensic Medicine, Kurume University School of Medicine, Kurume, Japan

*Corresponding Author

Kei Fukami

Division of Nephrology, Department of Medicine, Kurume University School of Medicine.

Address: 67 Asahi-machi, Kurume city, Fukuoka, Japan

Tel.: +81-942-31-7002; Fax: +81-942-31-7763.

E-mail: fukami@med.kurume-u.ac.jp

Supplementary Figure.1

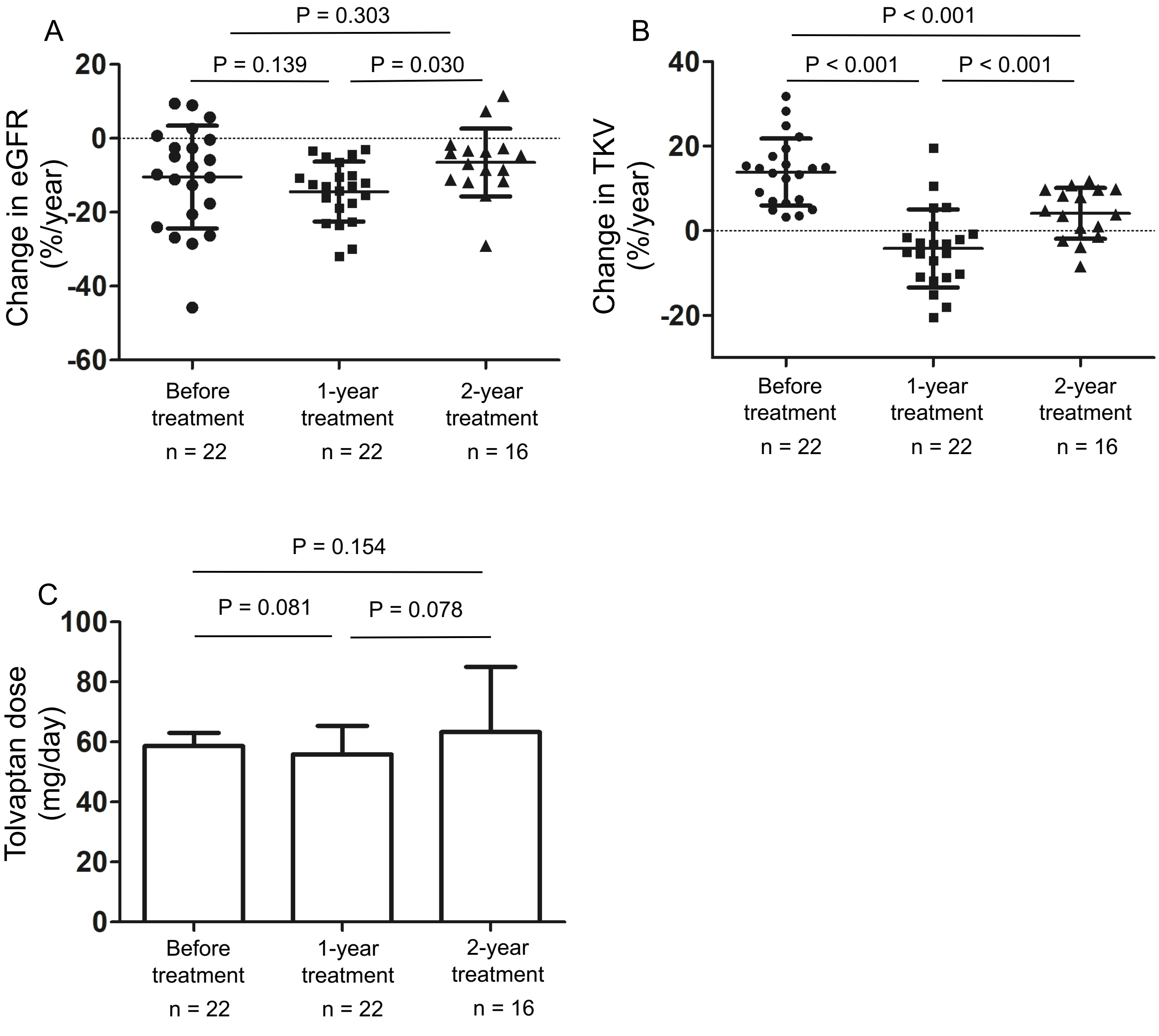


Effects of tolvaptan on change in eGFR and TKV 1-year before, 1-year after, and 2-year after the treatment. (A) eGFR 1-year before (n = 22), 1-year after (n = 22), and 2-year after (n = 16) the tolvaptan treatment. (B) Change in TKV 1-year before (n = 22), 1-year after (n = 22), and 2-year after (n = 16) the tolvaptan treatment. (C) Mean dose of tolvaptan 1-year before (n = 22), 1-year after (n = 22), and 2-year after (n = 16) the tolvaptan treatment.

eGFR: estimated glomerular filtration rate, TKV: total kidney volume.

# Supplementary Figure.2

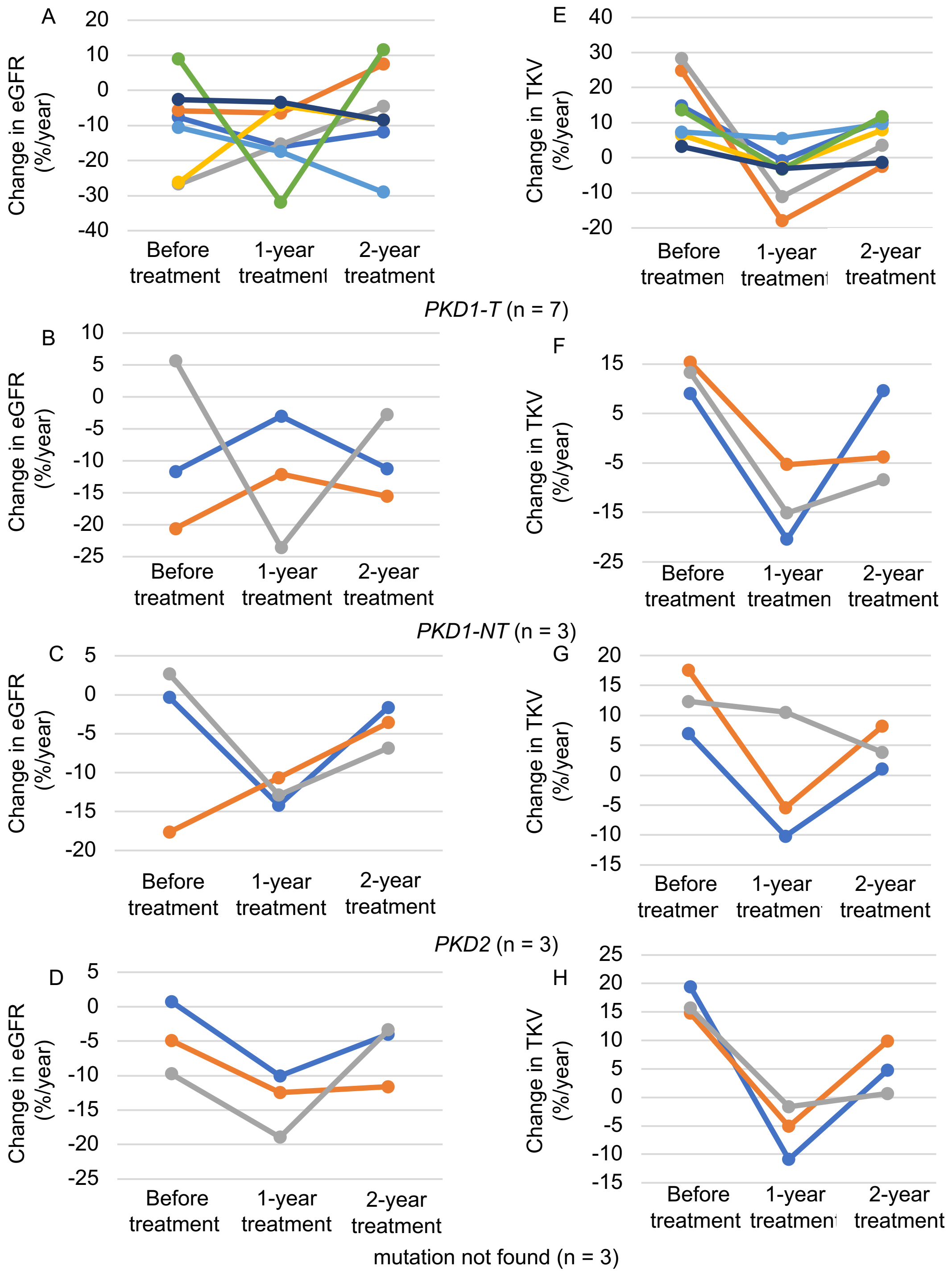


Effects of tolvaptan on changes in eGFR and TKV 1-year before, 1-year after, and 2-year after the treatment according to the gene mutation.

Change in eGFR 1-year before, 1-year after, 2-year after the tolvaptan treatment in patients with *PKD1-T* (A), *PKD1-NT* (B), *PKD2* (C), or mutation not found (D). Change of TKV 1-year before, 1-year after, 2-year after the tolvaptan treatment in patients with *PKD1-T* (E), *PKD1-NT* (F), *PKD2* (G), or mutation not found (H).

eGFR: estimated glomerular filtration rate, TKV: total kidney volume, *PKD1-T*: polycystic kidney disease1-truncating gene mutation, *PKD-NT*: PKD-non-truncating gene mutation.
